# Corrigendum: Lithium-ion-based solid electrolyte tuning of the carrier density in graphene

**DOI:** 10.1038/srep38054

**Published:** 2016-11-28

**Authors:** Jialin Zhao, Meng Wang, Hui Li, Xuefu Zhang, Lixing You, Shan Qiao, Bo Gao, Xiaoming Xie, Mianheng Jiang

Scientific Reports 6: Article number: 34816; 10.1038/srep34816 published online: 10 04 2016; updated: 11 28 2016

The Acknowledgements section in this Article is incorrect.

“We thank Chilin Li for providing the information of the solid electrolytes and Xiaoyu Liu for the help with nano-pattering. We acknowledge the support from the “Strategic Priority Research Program (B)” of the Chinese Academy of Sciences under Grant No. XDB04010600 and No. XDB04030000; from the National Natural Science Foundation of China under Grant No. 11374321; and from Helmholtz Association through the Virtual Institute for Topological Insulators (VITI)”.

Should read

“We acknowledge that the research was inspired by Prof. Xianhui Chen’s lecture in last November, in which Prof. Chen presented his application of solid electrolyte gating technique in the study of FeSe superconductivity, and that one of the contributing authors discussed with Prof. Chen about solid electrolyte gating after the lecture. We thank Chilin Li for providing the information of the solid electrolytes and Xiaoyu Liu for the help with nano-pattering. We acknowledge the support from the “Strategic Priority Research Program (B)” of the Chinese Academy of Sciences under Grant No. XDB04010600 and No. XDB04030000; from the National Natural Science Foundation of China under Grant No. 11374321; and from Helmholtz Association through the Virtual Institute for Topological Insulators (VITI)”.

