# Supplementary material for: Lithium-ion-based solid electrolyte tuning of the carrier density in graphene
Source: Sci Rep. 2016 Oct 4;6:34816. doi: 10.1038/srep34816 (PMC5048137; doi:10.1038/srep34816)
Supplement: Supplementary Information [file srep34816-s1.pdf]

Supplementary Materials for

Lithium-ion-based solid electrolyte tuning of the carrier density in graphene

Jialin Zhao[1,2], Meng Wang[1,2], Hui Li[3], Xuefu Zhang[1,2], Lixing You[1,2], Shan Qiao[1,2], Bo Gao[1,2], Xiaoming Xie[1,2], Mianheng Jiang[1,2,4]

[1]State Key Laboratory of Functional Materials for Informatics, Shanghai Institute of Microsystem and Information Technology, Chinese Academy of Sciences, 865 Changning Road, Shanghai 200050, China.

[2]CAS Center for Excellence in Superconducting Electronics (CENSE), Shanghai 200050, China

[3]Shenzhen Key laboratory of Laser Engineering, College of Optoelectronic Engineering, Shenzhen University, Shenzhen, 518060, China

[4]School of Physical Science and Technology, ShanghaiTech University, 319 Yueyang Road, Shanghai, China.

E-mail: bo_f_gao@mail.sim.ac.cn

This file includes:

Fig. S1 and Fig. S2

Fig. S1

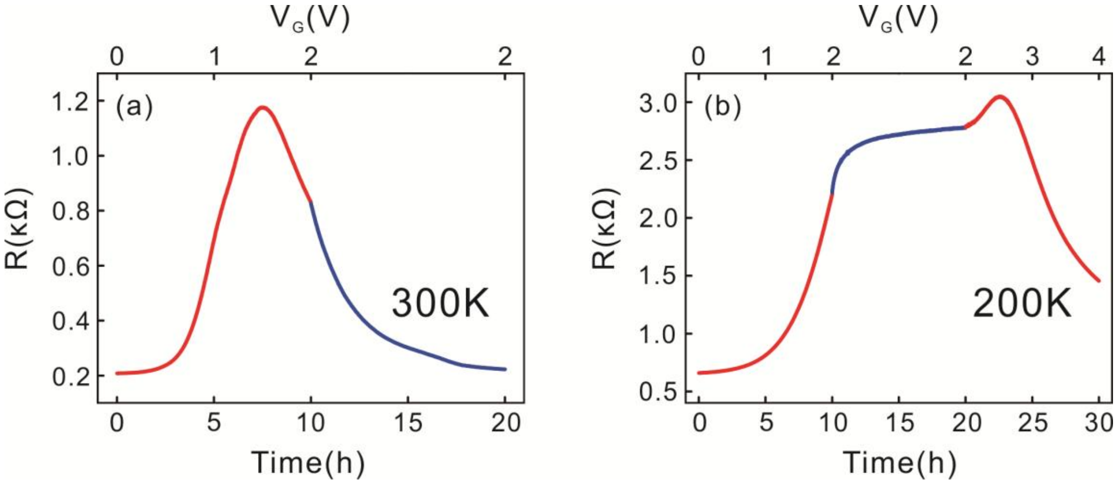

We carried out two experiments to demonstrate the evolution of Li-ions accumulation at the LICGC–graphene interface and the decline of the performance of the LICGC substrate in tuning the carrier density at low temperature. We used newly grown single-layer graphene whose resistance peak appear near 1.5 V (gate voltage sweep rate ~ 0.05 mV s^{-1}) at room temperature. Figure S1a shows the evolution the graphene resistance with time and applied gate voltage at room temperature. We swept the gate voltage from 0 to 2 V, as shown by the curve in red. We found that the resistance peak appeared near 1.5 V. We kept the back gate voltage at 2 V and recorded the change of the sample resistance with time, shown by the curve in blue. The resistance of the sample continued to drop, suggesting that the double layer capacitor was being charged. This is due to the long RC time constant. It explains the resistance discontinuity in Fig. 3a and 3d in the main text. Figure S1b shows similar measurements performed at 200 K. The gate voltage was first swept from 0 to 2 V. The resistance peak did not appear near 1.5V. If the only change at low temperature had been the enlarged RC time constant, we would expect that the resistance peak appear after a sufficiently long time. However, the resistance saturated after 10 hours. The resistance peak finally appeared after we swept the gate voltage to 3 V. This experiment suggests that the LICGC substrate shows weaker performance when used to tune the carrier density at low temperature. It loses completely the ability to tune the carrier density below 100 K, as shown in Fig. 4 in the main text. The possible explanation is that a higher back gate voltage may

be required to overcome the energy barrier encountered by lithium ions moving toward the LICGC–graphene interface at low temperature.

Fig. S2

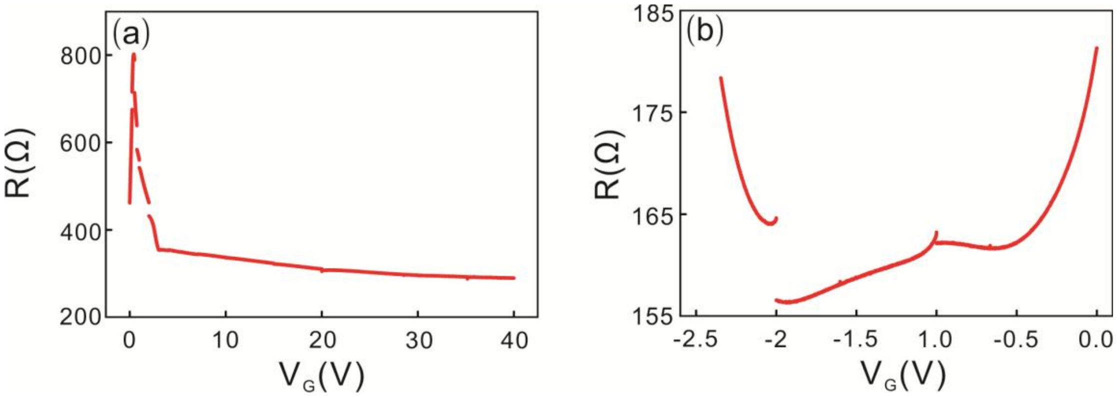

We also tried to apply high back gate voltage to investigate the performance of the LICGC solid electrolyte in such condition. We found that high positive gate voltage did not degrade significantly the graphene sample, while high negative gate voltage caused an abrupt rising of the sample resistance. The discontinuity in the above curves is due to the delayed Li ion displacements during the time interval in the measurements. It is known that electro-chemical reaction may take place when gating with liquid electrolytes. And sometimes it may bring additional electro-chemical doping effect that enhances the gate tuning effect [1,2]. We are investigating if similar electro-chemical doping also take places in the solid electrolyte gating process.

1 Li, Z. J., Gao, B. F., Zhao, J. L., Xie, X. M. & Jiang, M. H. Effect of electrolyte gating on the superconducting properties of thin 2H-$NbSe_2$ platelets. *Supercond. Sci. Tech* **27**, 015004, doi:10.1088/0953-2048/27/1/015004 (2014)..

2 Yu, Y. *et al.* Gate-tunable phase transitions in thin flakes of 1T-TaS_2.

Nat. Nanotechnol. **10**, 270-276, doi:10.1038/nnano.2014.323 (2015).
